# Home-Based vs Supervised Inpatient and/or Outpatient Rehabilitation Following Knee Meniscectomy: A Systematic Review and Meta-analysis

**DOI:** 10.1001/jamanetworkopen.2021.11582

**Published:** 2021-05-26

**Authors:** Sebastiano Nutarelli, Eamonn Delahunt, Marco Cuzzolin, Marco Delcogliano, Christian Candrian, Giuseppe Filardo

**Affiliations:** 1 Orthopaedic and Traumatology Unit, Ospedale Regionale di Lugano, EOC, Lugano, Switzerland; 2 School of Public Health, Physiotherapy and Sports Science, University College Dublin, Dublin, Ireland; 3 Institute for Sport and Health, University College Dublin, Dublin, Ireland; 4 USI-Università della Svizzera Italiana, Faculty of Biomedical Sciences, Lugano, Switzerland; 5 Applied and Translational Research Center, IRCCS Istituto Ortopedico Rizzoli, Bologna, Italy

## Abstract

**Importance:**

Arthroscopic meniscectomy is one of the most common orthopedic procedures. The optimal postoperative approach remains debated.

**Objective:**

To compare outcomes associated with home-based rehabilitation programs (HBP) vs standard inpatient and/or outpatient supervised physical therapy (IOP) following arthroscopic isolated meniscectomy (AM).

**Data Sources:**

A systematic literature search was conducted on PubMed, Web of Science, Cochrane Library, and Scopus databases on March 15, 2021. The included studies were published from 1982 to 2019.

**Study Selection:**

Randomized clinical trials of patients treated with HBP vs IOP after AM were included.

**Data Extraction and Synthesis:**

Data were independently screened and extracted by 2 authors according to the Preferred Reporting Items for Systematic Reviews (PRISMA) reporting guideline. The meta**-**analysis was performed using a random-effect model; when an *I*^2^ < 25% was observed, the fixed-effect model was used. The Hartung-Knapp correction was applied.

**Main Outcomes and Measures:**

The primary outcome was the Lysholm score (scale of 0-100 with higher scores indicating better knee function) and secondary outcomes were subjective International Knee Documentation Committee (IKDC) score, knee extension and flexion, thigh girth, horizontal and vertical hop test, and days to return to work, as indicated in the PROSPERO registration. Outcomes were measured in the short-term (ranging from 28 to 50 days) and the midterm (6 months).

**Results:**

In this meta-analysis of 8 RCTs including 434 patients, IOP was associated with a greater short-term improvement in Lysholm score compared with HBP, with a mean difference of −8.64 points (95% CI, −15.14 to −2.13 points; *P* = .02) between the 2 approached, but the sensitivity analysis showed no difference. Similarly, no statistically significant difference was detected at midterm for Lysholm score, with a mean difference between groups of −4.78 points (95% CI, −9.98 to 0.42 points; *P* = .07). HBP was associated with a greater short-term improvement in thigh girth, with a mean difference between groups of 1.38 cm (95% CI, 0.27 to 2.48 cm; *P* = .01), whereas IOP was associated with a better short-term vertical hop score, with a mean difference between groups of −3.25 cm (95% CI, −6.20 to −0.29 cm; *P* = .03). No differences were found for all the other secondary outcomes.

**Conclusions and Relevance:**

No intervention was found to be superior in terms of physical and functional outcomes as well as work-related and patient-reported outcomes, both at short-term and midterm follow-up. Overall, these results suggest that HBP may be an effective management approach after AM in the general population.

## Key Points

**Question:**

Is inpatient and/or outpatient rehabilitation associated with better recovery compared with home-based rehabilitation after arthroscopic isolated meniscectomy?

**Findings:**

This systematic review and meta-analysis compared home-based rehabilitation vs inpatient and/or outpatient rehabilitation after arthroscopic isolated meniscectomy, including 8 randomized clinical trials with a total of 434 participants. Inpatient and/or outpatient rehabilitation was not associated with better outcomes in terms of knee function (Lysholm score) at both short-term and midterm follow-up.

**Meaning:**

These findings suggest that home-based rehabilitation is a suitable option for recovery after arthroscopic isolated meniscectomy in the general population.

## Teaser

This meta-analysis examines 8 randomized clinical trials and assesses whether home-based rehabilitation or inpatient and/or outpatient rehabilitation is associated with better outcomes among patients recovering from arthroscopic isolated meniscectomy.

## Introduction

Meniscus injuries occur in physically active individuals, as well as members of the general population,^1,2,3,4^ with annual incidence rates of 66 to 70 per 100 000 persons reported.^5,6,7^ Meniscectomies are a primary risk factor for knee osteoarthritis,^8,9,10,11,12,13^ which led to efforts toward developing solutions to preserve or restore as much meniscal tissue as possible.^14,15,16,17,18^ Unfortunately, surgical intervention is not always avoidable, and arthroscopic isolated meniscectomy (AM) remains one of the most commonly performed orthopedic procedures.^19,20^

AM is a procedure in which a damaged meniscus is partially or completely removed. This entails a surgical trauma to the knee requiring postoperative management to facilitate the restoration of normal joint function. Numerous studies have investigated post-AM treatments during the previous decades.^21^ Nevertheless, the optimal postoperative approach is debated.^22^ Some authors investigated home-based rehabilitation programs (HBP) instead of standard inpatient and/or outpatient supervised physical therapy (IOP). In view of the high number of AM procedures performed globally, their societal impact and costs,^23,24^ and considering that HBP confers a cost reduction compared with IOP,^25^ understanding the potential and limitations of HBP-based AM postoperative management would be of substantial relevance for patients, physicians, and health care systems worldwide. Moreover, this is particularly relevant in the current COVID-19 pandemic scenario, whereby limiting the need for travel and personal contacts for face-to-face clinical consultations, including treatment exposure required by classical IOP approaches, is of utmost importance.^26,27,28^ The aim of this systematic review and meta-analysis was to compare the outcomes associated with HBP vs standard IOP after AM.

## Methods

### Search Strategy and Article Selection

The study protocol was registered in PROSPERO (CRD42020188377), and a systematic literature search was conducted on March 15, 2021, in PubMed, Web of Science, Cochrane Library, and Scopus using the following string: (*physical therapy* OR *physiotherapy* OR *rehabilitation* OR *exercise* OR *exercise therapy* OR *home exercise program* OR *home exercise therapy* OR *home exercise*) AND *meniscectomy*. Patients who underwent AM with postoperative HBT or IOP management were considered eligible. Duplicates were removed and records were screened for eligibility by title and abstract with whole text screening undertaken when required. The inclusion and exclusion criteria are described in eTable 1 in the Supplement. This meta-analysis followed the Preferred Reporting Items for Systematic Reviews and Meta-analyses (PRISMA) reporting guideline.^29^ The selection of studies was independently performed by 2 authors (S.N. and M.C.) with disagreements solved by consensus or by the intervention of a third author (M.D.) to assess the relevance of the articles which were considered then for the next step.

### Data Extraction, Synthesis, and Measurement of Outcomes

Data from the included studies were independently extracted by 2 authors (S.N. and M.C.) following Cochrane recommendations.^30^ Patients’ characteristics and clinical outcomes of treatments were extracted as follows: number of patients screened, included, and assessed at follow-up, patients’ presurgical and postsurgical Lysholm score (primary outcome) and subjective International Knee Documentation Committee (IKDC) score (both ranging from 0 to 100 with higher scores indicating fewer symptoms and disability), knee joint flexion and extension (degrees), thigh girth (centimeters), vertical and horizontal single-leg hop test (centimeters), and time to return to work in days (secondary outcomes). The outcome measures were classified as (1) patient-reported outcomes: Lysholm score, subjective IKDC score; (2) physical outcomes: knee flexion, knee extension, thigh girth; (3) functional outcomes: single hop test, vertical hop test; and (4) work-related outcomes: days needed to return to work. Details are reported in the Table. Missing information was requested by contacting the corresponding author of the relevant study.

**Table.** Characteristics of the Included Studies

Study	Intervention group [dropouts at last follow-up]	Control group [dropouts at last follow-up]	Treatment frequency and duration	Follow-up	Outcome measures	Difference of means (favors HBP or IOP)
Akkaya et al,^36^ 2012	EBT (group A) and NEMS + HBP (group B) combined for meta-analysis (n = 30) [none]	HBP (n = 15); duration: 1 mo [none]	I: 5 d/wk for the first 2 wk post-op; C: 1 mo	6 wk (short-term)	Lysholm score	6.2 (IOP)
				6 wk (short-term)	Knee flexion	8.2° (IOP)
				6 wk (short-term)	Knee extension	0.9° (HBP)
				6 wk (short-term)	Thigh girth	0.7 cm (HBP)
Forster and Frost,^23^ 1982	Outpatient PT + HBP (n = 44) [1]	HBP (n = 42) [1]	I: 3 times/wk for 4 wk, start on the 12th postop d (duration by clinical judgment); C: not specified	6 wk (short-term)	Knee flexion	0.9° (IOP)
				6 wk (short-term)	Thigh girth	0.4 cm (IOP)
				Absolute data	DRW	4 d (HBP)
Goodwin et al,^37^ 2003^a^	Supervised PT + HBP (n = 44) [5]	HBP (n = 40) [10]	I: 3 times/wk for 6 wk; C: 6 wk	50 d (short-term)	Single hop	11.9 cm (IOP)
				50 d (short-term)	Vertical hop	3.47 cm (IOP)
				Absolute data	DRW	12.6 d (HBP)
Hadley et al,^38^ 2019	Outpatient PT (n = 46) [not indicated]	Internet-based rehabilitation (n = 51) [not indicated]	I: 2 sessions/wk for 4-6 wk; C: minimum 3 times/wk (duration not specified)	6 wk (short-term)	Lysholm score	6.5 (IOP)
				6 mo (midterm)	Lysholm score	5.67 (IOP)
				6 wk (short-term)	IKDC	4.6 (IOP)
Kelln et al,^39^ 2009^b^	Active RoM recovery on a bicycle ergometer + HBP (n = 16) [2]	HBP (n = 15) [1]	I: 3 times/wk (duration not specified); C; duration not specified	1 mo (short-term)	IKDC	9.6 (IOP)
				1 mo (short-term)	Knee flexion	12.2° (IOP)
				1 mo (short-term)	Knee extension	0.5° (HBP)
				1 mo (short-term)	Thigh girth	1.9 cm (HBP)
Kirnap et al,^40^ 2005	EMG biofeedback training + HBP (n = 20) [none]	HBP (n = 20), duration 1 mo [none]	I + C: 5 times/wk for 2 wk	6 wk (short-term)	Lysholm score	15.8 (IOP)
				6 wk (short-term)	Knee flexion	7.9° (IOP)
Moffet et al,^41^ 1994	Outpatient PT + HBP (n = 15) [1]	HBP (n = 16) [none]	I + C: 3 wk	3 mo (short-term)	Lysholm score	3 (IOP)
				6 mo (midterm)	Lysholm score	2 (IOP)
Vervest et al,^42^ 1990	Exercise-based outpatient PT (n = 10) [none]	HBP + verbal and written advice (n = 10) [none]	I: 30-min sessions for 3 wk; C: duration not specified	28 d (short-term)	Lysholm score	9.3 (IOP)
				28 d (short-term)	Single hop	19.1 cm (IOP)
				28 d (short-term)	Vertical hop	2.4 cm (IOP)

Abbreviations: C, control group; DRW, days to return to work; EBT, electromyography biofeedback therapy; HBP, home-based program; I, intervention group; IKDC, subjective International Knee Documentation Committee score; IOP, inpatient and/or outpatient rehabilitation; NEMS, neuromuscular electrical stimulation; PT, physical therapy; RoM, range of motion.

^a^ Data retrieved contacting the authors.

^b^ Data extracted from plots via WebPlotDigitizer version 4.3 (Ankit Rohatgi).

### Statistical Analysis

The risk of bias of each included study was evaluated using version 2 of the Cochrane risk-of-bias tool for randomized trials.^31^ The overall quality of evidence for each outcome was rated according to the Grading of Recommendations Assessment, Development, and Evaluation (GRADE) guidelines.^32^ The statistical analysis of primary and secondary outcomes was performed to compare HBP and IOP effectiveness following isolated AM. When data from the same study population were available at different follow-ups, the closest to 6 weeks were selected for the short-term evaluation. A separate midterm analysis was performed after 3 months. In all cases, the actual point in time scores were meta-analyzed. The inverse variance method for continuous variables was used to measure the difference between the outcome measures with results expressed as mean differences (MD). Heterogeneity was tested using *I*^2^ metric and considered significant when *I*^2^ > 25%. As recommended by the article by Borenstein et al,^33^ the meta-analysis was performed using a random-effect model under the assumption that significant differences among studies could not justify a fixed-effect model. As such, when an *I*^2^ < 25% was observed, the meta-analysis was reimplemented applying a fixed-effect model. A *P* value of .05 was set as the level of significance for the analysis with 2-sided testing. The Hartung-Knapp correction^34^ was applied to properly analyze the outcomes generated by few articles. When means and standard deviations were not reported in the included studies, they were obtained from medians and ranges with the estimation method proposed by Wan et al^35^ following the Cochrane guidelines.^30^ The statistical analysis was performed using R software version 1.2.5019 (R Project for Statistical Computing) with the meta (version 4.9-7), dmetar (version 0.0.9000), and metafor (version 2.1-0) packages in March 2021.

## Results

### Study Selection and Patients’ Characteristics

**Figure 1.**
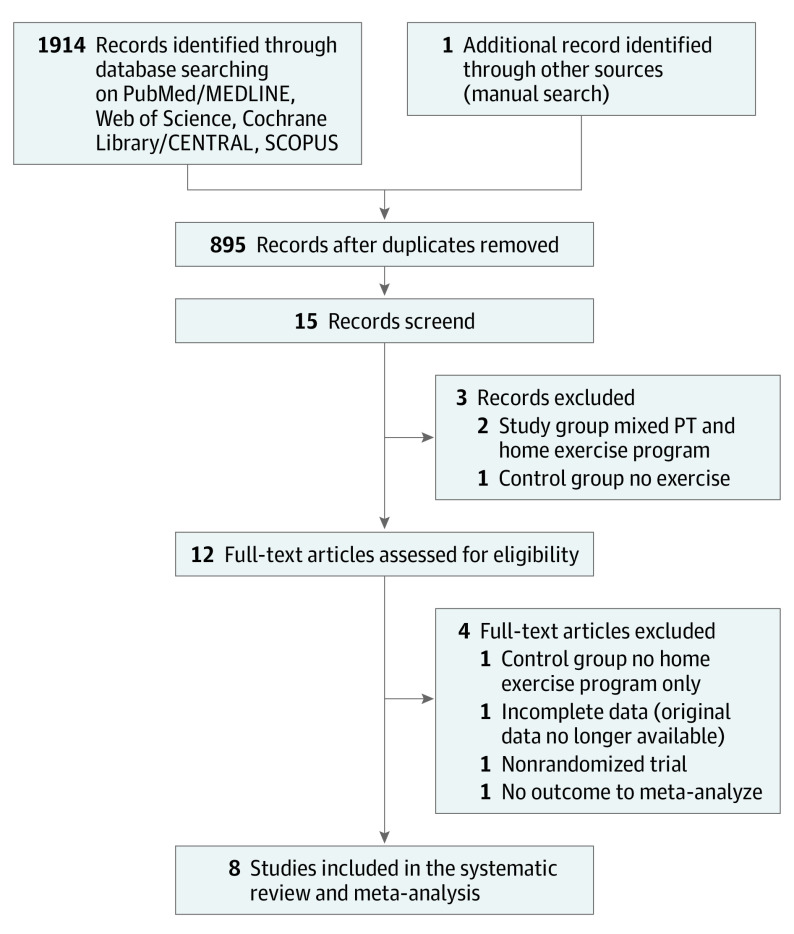
Flowchart of the Study Selection Process PT indicates physical therapy.

**Figure 2.**
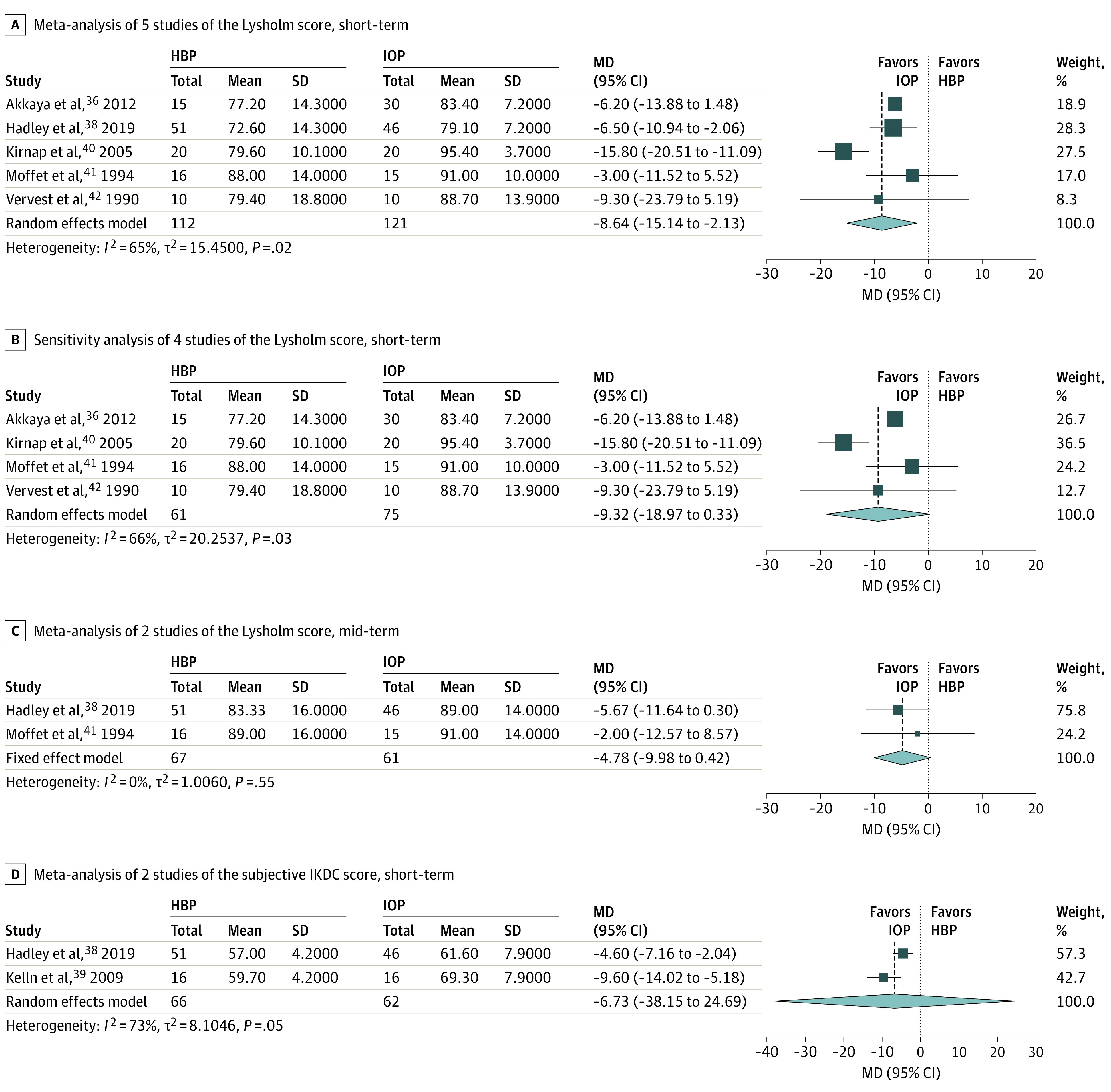
Forest Plots of the Patient-Reported Outcomes The outcomes of home-based rehabilitation (experimental group) are compared with inpatient and/or outpatient rehabilitation (control group) and the performed sensitivity analysis. Lysholm scores and IKDC scores range from 0 to 100 with higher scores indicating better knee function and fewer symptoms. Values for mean, SD, and MD Lysholm and IKDC scores are given as points. HBP indicates home-based rehabilitation; IKDC, subjective International Knee Documentation Committee score; IOP, inpatient and/or outpatient rehabilitation; MD, mean difference.

**Figure 3.**
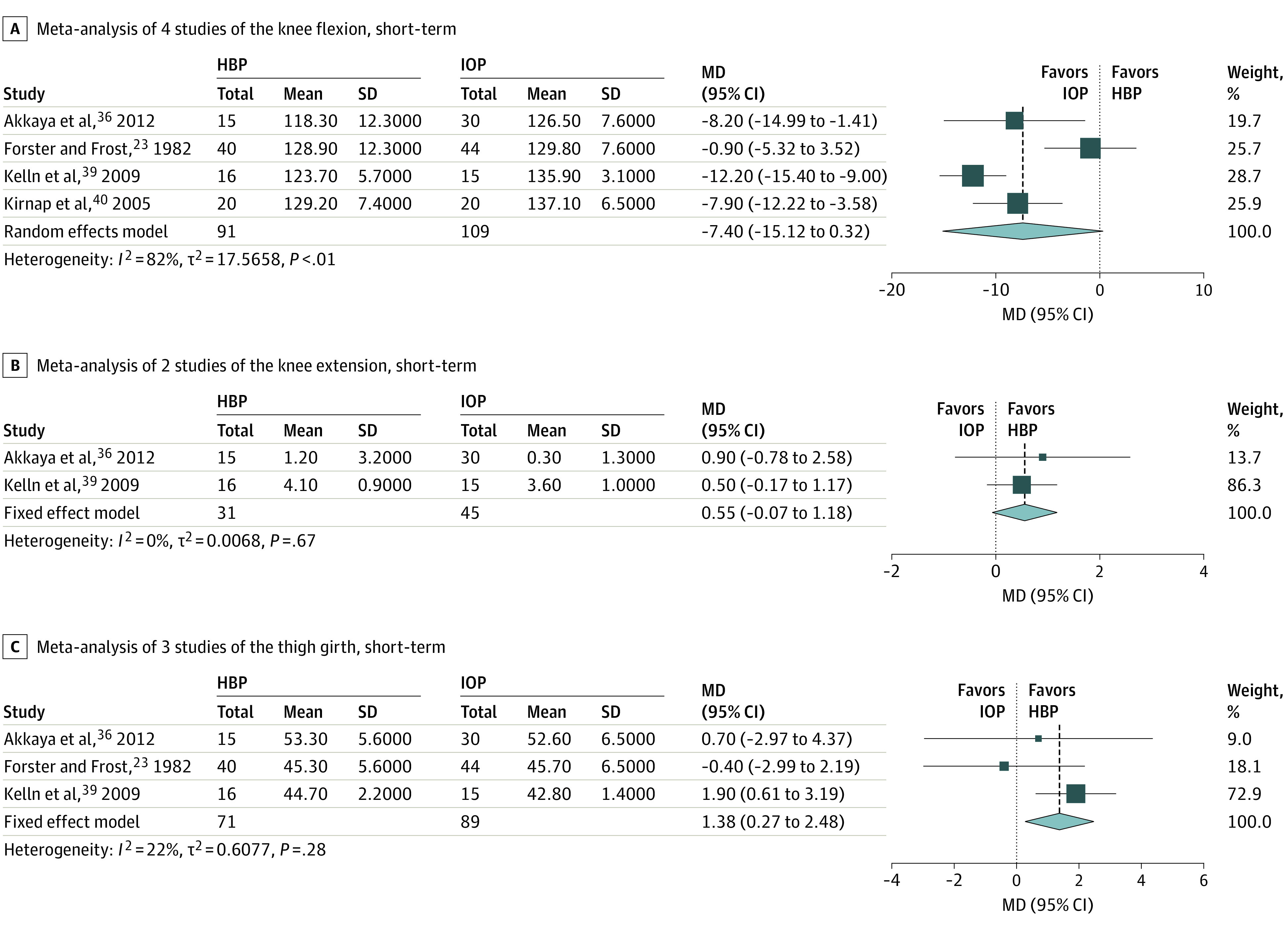
Forest Plots of the Physical Outcomes The outcomes of home-based rehabilitation (experimental group) are compared with inpatient and/or outpatient rehabilitation (control group). Values for mean, SD, and MD knee flexion and knee extension are given as degrees. Values for mean, SD, and MD thigh girth are given as centimeters. HBP indicates home-based rehabilitation; IOP, inpatient and/or outpatient rehabilitation; MD, mean difference.

**Figure 4.**
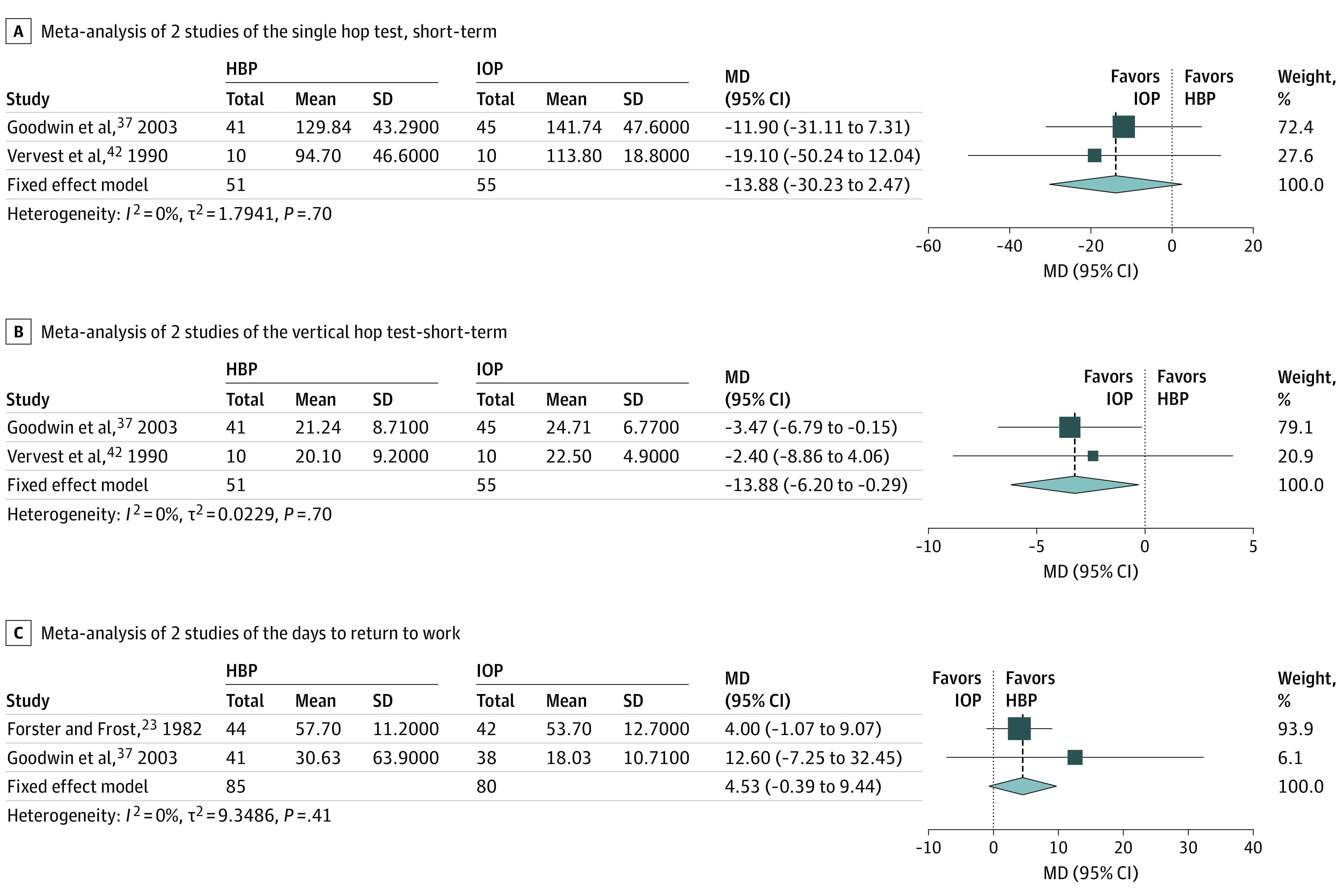
Forest Plots of the Functional and Work-Related Outcomes The outcomes of home-based rehabilitation (experimental group) are compared with inpatient and/or outpatient rehabilitation (control group). Values for mean, SD, and MD single hop and vertical hop tests are given as centimeters. Values for total, mean, and SD return to work are given as days. HBP indicates home-based rehabilitation; IOP, inpatient and/or outpatient rehabilitation; MD, mean difference.

The flowchart of the article selection process is reported in Figure 1. Out of the 1914 records retrieved, 8 studies were included^23,36,37,38,39,40,41,42^ and reported data that could be aggregated to be analyzed via meta-analysis (Figure 2, Figure 3, Figure 4). All studies selected were RCTs published from 1982 to 2019 reporting on 434 individuals (age range: 21 to 74 years) and comparing HBP (209 participants) vs IOP (225 participants) after AM. All studies reported the participants’ gender, with an overall distribution of 332 men and 104 women. In one case where the data were published in a ratio fashion, the authors provided the original data to be included in the study.^37^

The IOP approach differed in the included studies in terms of interventions provided, but they all consisted of standard inpatient and/or outpatient supervised rehabilitation sessions (1:1), primarily comprising exercises with the addition of modalities^37,41^ such as EMG biofeedback training (EBT),^40^ EBT and neuromuscular electrical stimulation,^36^ soft-tissue treatments and manual therapy,^37^ and isokinetic training.^41^ The HBPs in the included studies were characterized by verbal and written with sometimes illustrated indications of the exercises. In one study, composed solely of an abstract with outcome-related data in a tabular format, HBP was delivered via internet-based rehabilitation.^38^ The follow-up length ranged from 28 days to 6 months. Details are reported in the Table.

### Patient-Reported Outcomes 

#### Lysholm Score

Meta-analysis of 5 studies showed an initial between-groups difference at short-term (range: 28 days to 3 months) favoring IOP; the MD was −8.64 points (95% CI, −15.14 to −2.13 points; *P* = .02). The greatest MD across the studies was 15.8 points (Figure 2A). Given that one paper included was a published abstract providing data in a tabular format,^38^ we performed a sensitivity analysis to ascertain the robustness of the meta-analysis outcome. Hence the primary meta-analysis was repeated restricting the analysis to the other included studies. The results differed from the original pooled-effect analysis displaying no difference (Figure 2B).

Meta-analysis of 2 studies did not show a between-groups difference at midterm after 6 months. The MD was −4.78 points (95% CI,−9.98 to 0.42 points; *P* = .07); the greatest MD found was 5.67 points (Figure 2C).

#### Subjective IKDC Score

Meta-analysis of 5 studies did not show a between-groups short-term difference (range: 1 month to 6 weeks). The MD was −6.73 points (95% CI, −38.15 to 24.69 points; *P* = .22). The greatest MD found was 9.6 points (Figure 2D).

### Physical Outcomes

#### Knee Flexion and Extension

Meta-analysis of 4 studies did not show a between-groups short-term difference (range: 1 month to 6 weeks) in knee flexion; the MD was −7.40° (95% CI, −15.12° to 0.32°; *P* = .055). The greatest MD found was 12.2° (Figure 3A). Meta-analysis of 2 studies did not show a between-groups short-term difference (range: 1 month to 6 weeks) in knee extension; the MD was 0.55° (95% CI, −0.07° to 1.18°; *P* = .08). The greatest MD found was 0.9° (Figure 3B).

#### Thigh Girth

Meta-analysis of 3 studies showed a between-groups short-term difference (range: 1 month to 6 weeks) in thigh girth favoring HBP; the MD was 1.38 cm (95% CI, 0.27 to 2.48 cm; *P* = .01). The greatest MD found was 1.9 cm (Figure 3C).

### Functional Outcomes

#### Single Hop Test

Meta-analysis of 2 studies did not show a between-groups short-term difference (range: 28 to 50 days); the MD was −13.88 cm (95% CI, −30.23 to 2.47 cm; *P* = .10). The greatest MD found was 19.1 cm (Figure 4A).

#### Vertical Hop Test

Meta-analysis of 2 studies showed a between-groups short-term difference (range: 28 to 50 days), which favored IOP; the MD was −3.25 cm (95% CI, −6.20 to −0.29 cm; *P* = .03). The greatest MD found was 3.47 cm (Figure 4B).

### Work-Related Outcome

Meta-analysis of 2 studies did not show a between-groups difference in time to return to work; the MD was 4.53 days (95% CI, −0.39 to 9.44 days; *P* = .07). The greatest MD found was 12.6 days (Figure 4C).

### Risk of Bias and Level of Evidence

The risk of bias assessment was conducted by 2 independent reviewers (S.N. and M.C.); the interrater reliability (κ = 0.913) disagreement was solved by consensus. The assessment resulted in some concerns of risk of bias in 4 studies^36,37,40,42^ and high risk of bias in 4 other studies.^23,38,39,41^ The main reasons were the absence of intention-to-treat analyses, lack of assessor blinding, missing detailed information in the published papers, and absence of indications of preregistered study protocols that might result in selective reporting bias risk. Visualizations of the risk of bias assessment results are detailed in the eFigure in the Supplement, produced with the Risk of Bias Visualization Online Tool.^43^ A risk-of-bias detailed table is available in eTable 2 in the Supplement providing further information on the performed assessment. The GRADE evidence profile for all the plotted outcomes resulted in low to very low and is reported in eTable 3 in the Supplement, generated via the GRADEpro online Guideline Development Tool (GDT).^44^

## Discussion

This systematic review and meta-analysis compared the outcomes of HBP vs standard IOP following isolated AM. No overall difference was documented in either the short-term or midterm across patient-reported outcomes, physical and functional outcomes, and work-related outcomes.

The retrieved data deserve a critical analysis. In fact, the first evaluation showed a short-term difference in terms of Lysholm score favoring IOP. However, the sensitivity analysis did not confirm this finding, underlying the importance of having high-level studies to investigate this issue. Moreover, the absolute score values did not differ also considering the minimal detectable change defined as 10.1 points for the Lysholm score when investigating meniscus injuries.^45^ A difference was instead shown in the thigh girth, favoring HBP, whereas a difference was found in the vertical hop test score, favoring IOP. Still, clinical meaningfulness of these differences is likely negligible. The overall overlapping benefit of IOP and HBP is particularly interesting considering the ongoing global COVID-19 pandemic. The travel restrictions and social distancing measures implemented worldwide to contain the spread of the virus highlight the importance of reducing unnecessary face-to-face clinical consultations and treatments, as well as overall people exposure.^46^ Furthermore, implementing HBP should be seen with favor considering the postoperative driving limitations of patients living in rural areas with no convenient access to physical therapy (PT) facilities,^24^ and should be pursued and further developed to optimize patient management after AM.

The search for the optimal post-AM management has been under investigation for almost 40 years. In 1989, Jokl et al^24^ reported no differences between supervised outpatient rehabilitation and HBP across a range of subjective and objective outcome measures, and the HBP group even showed a tendency to perform better in all the strength, power, and endurance isokinetic assessments with a quicker return to daily activities, work, and sport compared with IOP. The author was contacted but the original data needed for inclusion of this study were no longer retrievable. Also, Birch et al^47^ compared IOP and HBP, reporting no difference in outcome measures. Their study was not included because none of the outcome measures aligned with those of the meta-analysis protocol. Even though these studies could not be included, altogether the literature underlines no overall benefits of IOP over HBP. In this regard, it is also worth mentioning that Han et al^48^ found no difference comparing the outpatient PT with HBP even following a considerably more invasive procedure such as total knee replacement. However, advantages of IOP on post-AM rehabilitation were still underlined by some studies,^40,42^ and a faster functional recovery could be relevant especially for competitive athletes. The 2 functional outcomes included in this meta-analysis were measured within a follow-up range from 28 to 50 days, which lines up with the return to sport following AM.^49^ In this light, the vertical hop test favoring IOP and suggesting a benefit in terms of recovery time deserve to be better analyzed. The retrieved weighted MD was of only 3.25 cm. Moreover, this test has no established reliability or measurement error as assessed by the minimal important change or smallest detectable change.^50^ Therefore, the actual clinical significance of the intergroup difference emerged in this meta-analysis should be considered critically for the average patient. Further studies with subclassification of participants would empower an Evidence-Based Practice (EBP) targeted to specific patient populations.

“Patients are often prescribed PT after arthroscopy in the belief that knee function will be regained more quickly,”^47^ but thus far the published literature offers heterogeneous findings. Regarding this subject, it is worth pointing out that the RCTs analyzed present heterogeneous study methodology; thus the analysis implied a simplification of a complex field, and some aspects of the post-AM management emerged that deserve critical consideration and deeper research to further clarify this topic. For instance, Di Paola^51^ investigated the effect of a protocol-driven HBP compared with traditional outpatient PT in participants following AM and receiving workers’ compensation. The HBP group was also given a written referral with a predetermined maximum number of approved PT sessions to attend if needed, likely representing a deviation from a truly HBP approach. Because of the retrospective study design and contamination of the HBP treatment, this study was excluded from the meta-analysis. However, the study found no difference in time to release to light and full duty at work, time to claim closure, or rate of impairment and permanent disability rate. Interestingly, the number of attended PT sessions resulted 40% lower in the HBP group showing the way for an effective post-AM management with an associated marked cost-reduction. The author concluded that “Providing more services does not necessarily ensure better results and may have either no effect or a negative effect on functional or financial outcomes.”^51^ This issue has already been alluded to by Forster and Frost^23^ who indicated that since no difference was found between IOP and HBP “the resources saved by discontinuing routine PT after AM could be diverted to the rehabilitation of conditions in which benefit might accrue.” Jokl et al^24^ also came to the same conclusion while additionally illustrating that IOP is up to 21 times more expensive than HBP, with an average cost of $850.00 for IOP compared with $40.00 for HBP (this study was published in 1989, thus the absolute numbers no longer relate with the current economy). Di Paola highlighted that an optimized HBP approach would include the addition of a monitoring system, whereby the participants receive indications on expected subjective and functional outcomes, and specific objective weekly goals, the failure of meeting which “alerted the clinicians to the potential need for modification to the HBP regimen.”^51^ Such an approach was suggested by Jokl et al^24^ over 30 years ago, who contested that not all meniscectomized patients perform equally following surgery. Periodical follow-ups can identify who is not adequately progressing and should switch to IOP, embodying an optimized management. As this study concluded, most of the post-AM management could be alleviated by prescribing a properly monitored HBP.

HBP showed to be a suitable option in musculoskeletal rehabilitation,^52^ but factors such as patients’ perspectives and previous experiences should not be overlooked since they are symbiotically tied to program compliance and adherence hence fostering better prognosis.^53^ Many post-AM patients can develop over-dependency on supervised PT owing to their lack of knowledge, confidence, and equipment. However, the equipment needed to implement HBP is usually minimal, and a detailed exercise protocol outlining the treatment philosophy and clearly related goals can enhance patient adherence.^51^ Yilmaz et al^54^ illustrated that home exercises taught by a physical therapist were more useful for patients than an exercise leaflet alone among a group of patients with knee osteoarthritis, highlighting how patients’ education and coaching are important aspects when prescribing HBPs. Two systematic reviews^55,56^ investigated factors associated with a higher adherence to HBP, which found high self-efficacy and motivation, internal locus of control, limited feeling of helplessness, social support, positive feedback from a physical therapist, supervision, time-convenience, cost-reduction, and recurring to an exercise diary. In addition, a qualitative study of Palazzo et al^57^ documented patients' expectations regarding adherence with new technologies applied to the delivery of HBP, underlining that regardless of the proposed tool, patients expected to learn its use through a supervised session and their home performance regularly checked by health care practitioners, thus asking for some level of monitoring associated with HBP.

HBPs can be optimized by considering these findings. To this regard, Hadley et al^38^ investigated HBP delivered through an internet-based exercise program with no human interaction.^38^ Patients could message questions obtaining written replies by physical therapists. The login frequency and time spent watching the exercises videos suggested high adherence. Similarly, Russell et al^58^ found internet-based rehabilitation to be as effective as IOP in patients following total knee replacement. This approach appears to be inherently capable of enhancing adherence by addressing some of the previously mentioned barriers to HBP,^59^ ensuring supervision from a clinician, and is time- and money-saving for both health care systems and patients compared with standard IOP.^60^ Internet-based rehabilitation shares a lot in common with tele-rehabilitation, which provides additional support and feedback through onscreen face-to-face human interaction and proved noninferior to IOP after hospital discharge even in patients following markedly more invasive knee surgical procedures.^61^ This approach was found to be similar to face-to-face PT in terms of pain, function, and quality of life, concurrently matching patients’ satisfaction,^62^ being cost-effective,^63^ and reducing traveling time, costs, and work absenteeism associated with in-person appointments.^64^

These findings add to the present meta-analysis, which was conceived to compare outcomes associated with HBP vs IOP following AM, offering a comprehensive and transparent snapshot of the available scientific literature on this topic. This meta-analysis was able to provide a valuable clinical indication showing the overall nonsuperiority of IOP vs HBP. These results, together with other findings of the available literature, suggest the benefit of HBP and the possibility to further optimize a more balanced, combined approach that might express the highest potential, such as a monitored HBP with IOP mainly for the patients not progressing as expected. Tele- and internet-based rehabilitation could represent an effective way to monitor HBP, improving treatment adherence and the results of patients after AM.

### Limitations

There are multiple limitations to this meta-analysis. The number of included studies was low. The number of studies per outcome that concurred to the pooled data for the meta-analysis was low due to the heterogeneity of the outcomes retrieved in the specific literature; although the Hartung-Knapp correction was applied to address this limitation and properly analyze the outcomes, more high-level trials are needed to confirm the study findings. Only one midterm outcome was recorded (patient-reported outcome) limiting the overall strength of the observations at this time point. The inclusion of only RCTs elevated the level of this meta-analysis, but their risk of bias was assessed from some concerns to high and the low to very low GRADE evidence profile represent a limitation.

## Conclusions

The meta-analysis comprised the available RCTs in the scientific literature comparing HBP with IOP after AM. Overall, no intervention was found to be superior in terms of physical, functional, work-related, and patient-reported outcomes, both at short-term and midterm follow-ups. Thus, these findings suggest that HBP may be an effective management after AM in the general population.

## Notes

Supplement. **eFigure.** The Robvis Visual Summary of the Performed Risk of Bias Assessment **eTable 1.** Inclusion and Exclusion Criteria for Study Selection **eTable 2.** Risk of Bias Detailed Table **eTable 3.** GRADE Evidence Profile eReferences
