# Supplementary material for: Home-Based vs Supervised Inpatient and/or Outpatient Rehabilitation Following Knee Meniscectomy: A Systematic Review and Meta-analysis
Source: JAMA Netw Open. 2021 May 26;4(5):e2111582. doi: 10.1001/jamanetworkopen.2021.11582 (PMC8155825; doi:10.1001/jamanetworkopen.2021.11582)
Supplement: Supplement. — eFigure. The Robvis Visual Summary of the Performed Risk of Bias Assessment eTable 1. Inclusion and Exclusion Criteria for Study Selection eTable 2. Risk of Bias Detailed Table eTable 3. GRADE Evidence Profile eReferences [file jamanetwopen-e2111582-s001.pdf]

Supplemental Online Content

Nutarelli S, Delahunt E, Cuzzolin M, Delcogliano M, Candrian C, Filardo G. Home-based vs supervised inpatient and/or outpatient rehabilitation following knee meniscectomy: a systematic review and meta-analysis. *JAMA Netw Open.* 2021;4(5):e2111582. doi:10.1001/jamanetworkopen.2021.11582

eFigure. The Robvis Visual Summary of the Performed Risk of Bias Assessment

eTable 1. Inclusion and Exclusion Criteria for Study Selection

eTable 2. Risk of Bias Detailed Table

eTable 3. GRADE Evidence Profile

eReferences

This supplementary material has been provided by the authors to give readers additional information about their work.

© 2021 Nutarelli S et al. *JAMA Network Open.*

eFigure. The robvis visual summary of the performed risk of bias assessment

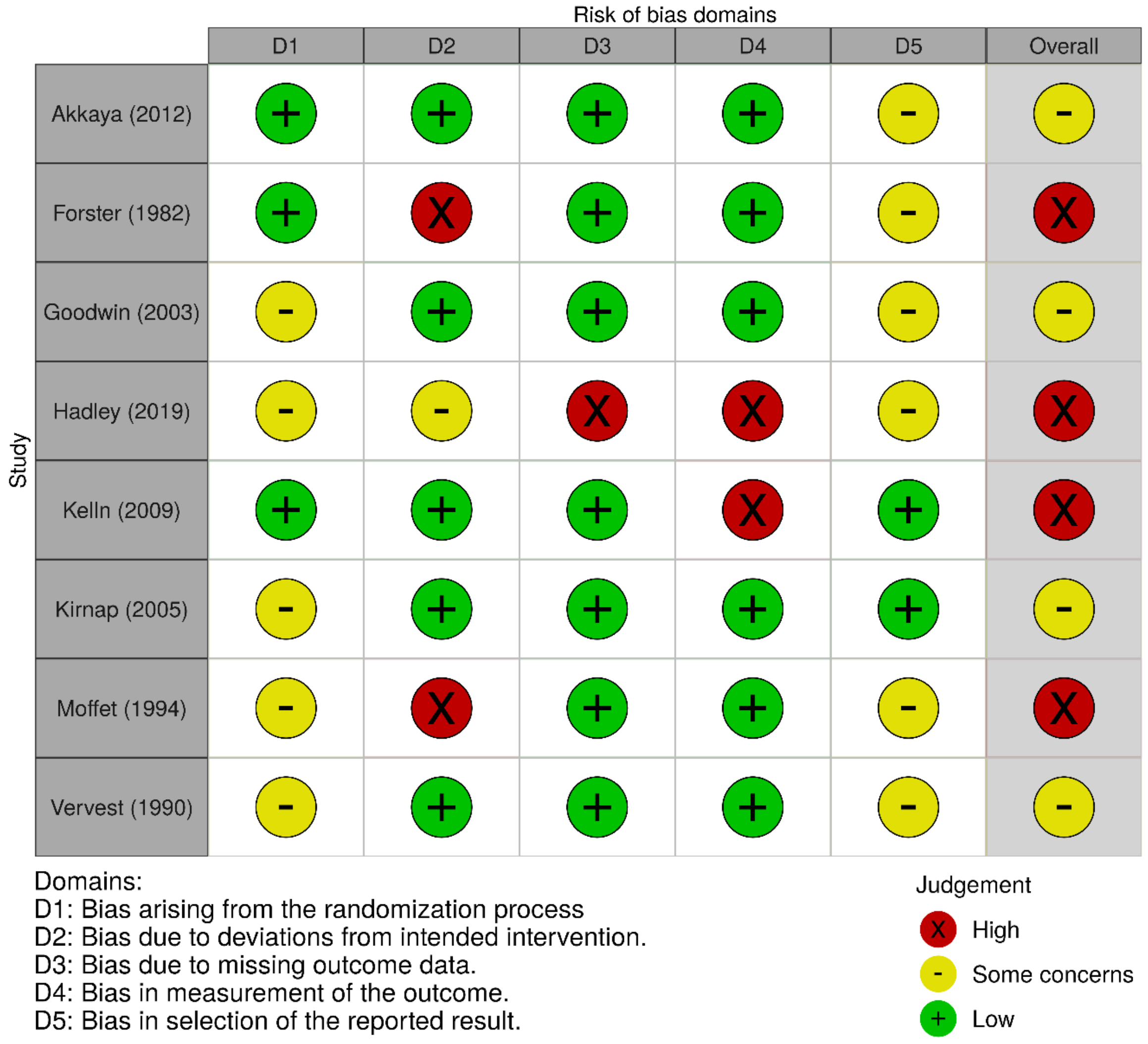

Note: RoB assessment differs across outcomes. Because the assessors were aware of intervention in all the included studies, the per outcome RoB should be rated higher for those outcomes rated by an assessor, such as the physical and functional outcomes. This does not affect the RoB of the PROs and work-related outcomes. The only included study providing outcomes not rated by an assessor (PROs) was the Moffet et al. (1994) study, therefore ensuring a lower per outcome RoB.

© 2021 Nutarelli S et al. *JAMA Network Open.*

eTable 1. Inclusion and exclusion criteria for study selection.

Inclusion criteria	Exclusion criteria
Studies comparing patients that underwent isolated AM and were treated with home-based PT versus inpatient and/or outpatient PT	Non-comparative studies
Randomized controlled trials	Non-randomized observational studies, expert opinions, systematic reviews, and meta-analyses
Human studies	Pre-clinical or *ex vivo* studies

Abbreviations: **AM**, Arthroscopic Meniscectomy; **PT**, Physical Therapy.

© 2021 Nutarelli S et al. *JAMA Network Open.*

eTable 2. Risk of bias detailed table

RoB 2.0	Overall	Randomization process (D1)				
**Study**	**Risk of bias**	**(1.1) Allocation sequence random?**	**(1.2) Allocation sequence concealed?**	**(1.3) Baseline imbalances suggest a problem?**	**(D1) Risk Of Bias**	**Direction**
Akkaya (2012)	SOME CONCERNS	Y	Y	N	LOW RISK	UNPREDICTABLE
Forster (1982)	HIGH RISK	Y	Y	N	LOW RISK	NA
Goodwin (2003)	SOME CONCERNS	Y	NI	PN	SOME CONCERNS	UNPREDICTABLE
Hadley (2019)	HIGH RISK	Y	NI	NI	SOME CONCERNS	UNPREDICTABLE
Kirnap (2005)	SOME CONCERNS	Y	NI	PN	SOME CONCERNS	UNPREDICTABLE
Kelln (2009)	HIGH RISK	Y	Y	NI	LOW RISK	UNPREDICTABLE
Moffet (1994)	HIGH RISK	Y	NI	PN	SOME CONCERNS	UNPREDICTABLE
Vervest (1990)	SOME CONCERNS	Y	NI	N	SOME CONCERNS	UNPREDICTABLE

© 2021 Nutarelli S et al. *JAMA Network Open.*

eTable 2-continues. Risk of bias detailed table

RoB 2.0	Effect of assignment to intervention (D2)								
**Study**	**(2.1) Participants aware of intervention?**	**(2.2) Personnel aware of intervention?**	**(2.3) Deviations arose because of the trial context?**	**(2.4) Deviations affect the outcome?**	**(2.5) Deviations balanced between groups?**	**(2.6) Appropriate analysis to estimate the effect of assignment (ITT)?**	**(2.7) Substantial impact of failure to analyze participants in randomized groups?**	**(D2) Risk of Bias**	**Direction**
Akkaya (2012)	Y	Y	PN	NA	NA	Y	NA	LOW RISK	UNPREDICTABLE
Forster (1982)	Y	Y	PN	NA	NA	N	PN	SOME CONCERNS	FAVORS EXPERIMENTAL
Goodwin (2003)	Y	Y	PN	NA	NA	Y	NA	LOW RISK	UNPREDICTABLE
Hadley (2019)	Y	Y	NI	NI	NI	NI	PN	SOME CONCERNS	UNPREDICTABLE
Kirnap (2005)	Y	Y	PN	NA	NA	PY	NA	LOW RISK	UNPREDICTABLE
Kelln (2009)	Y	Y	PN	NA	NA	Y	NA	LOW RISK	NA
Moffet (1994)	Y	Y	PN	NA	NA	N	PY	HIGH RISK	UNPREDICTABLE
Vervest (1990)	Y	Y	PN	NA	NA	PY	NA	LOW RISK	NA

© 2021 Nutarelli S et al. *JAMA Network Open.*

eTable 2-continues. Risk of bias detailed table

RoB 2.0	Effect of missing outcome data (D3)					
**Study**	**(3.1) Outcome data for all participants?**	**(3.2) Evidence that result is not biased?**	**(3.3) Missingness could depend on true value?**	**(3.4) Likely that missingness depended on true value?**	**(D3) Risk of bias**	**Direction**
Akkaya (2012)	Y	NA	NA	NA	LOW RISK	NA
Forster (1982)	Y	NA	NA	NA	LOW RISK	NA
Goodwin (2003)	Y	NA	NA	NA	LOW RISK	NA
Hadley (2019)	NI	NI	NI	NI	HIGH RISK	UNPREDICTABLE
Kirnap (2005)	Y	NA	NA	NA	LOW RISK	UNPREDICTABLE
Kelln (2009)	N	Y	NA	NA	LOW RISK	UNPREDICTABLE
Moffet (1994)	N	Y	NA	NA	LOW RISK	UNPREDICTABLE
Vervest (1990)	PY	NA	NA	NA	LOW RISK	NA

© 2021 Nutarelli S et al. *JAMA Network Open.*

eTable 2-continues. Risk of bias detailed table

RoB 2.0	Risk in the measurement of the outcome (D4)						
**Study**	**(4.1) Method of measuring the outcome inappropriate?**	**(4.2) Measurement of ascertainment of outcome differ between groups?**	**(4.3) Outcome assessors aware of intervention received?**	**(4.4) Could assessment have been influenced by knowledge of intervention?**	**(4.5) Likely that assessment was influenced by knowledge of intervention?**	**(D4) Risk of bias**	**Direction**
Akkaya (2012)	N	N	N	NA	NA	LOW RISK	NA
Forster (1982)	N	N	Y	PN	PY	LOW RISK	FAVORS THE EXPERIMENTAL
Goodwin (2003)	N	PN	N	NA	NA	LOW RISK	NA
Hadley (2019)	N	PN	NI	NI	NI	HIGH RISK	UNPREDICTABLE
Kirnap (2005)	N	PN	NI	PN	NA	LOW RISK	UNPREDICTABLE
Kelln (2009)	PY	PY	Y	NI	PY	HIGH RISK	UNPREDICTABLE
Moffet (1994)	N	PN	N	NA	NA	LOW RISK	NA
Vervest (1990)	N	PN	N	NA	NA	LOW RISK	NA

eTable 2-continues. Risk of bias detailed table

© 2021 Nutarelli S et al. *JAMA Network Open.*

RoB 2.0	Risk in the selection of the reported results (D5)				
**Study**	**(5.1) Trial analyzed in accordance with a pre-specified plan?**	**(5.2) Result selected from multiple outcome measurements?**	**(5.3) Result selected from multiple analysis of the data?**	**(D5) Risk of bias**	**Direction**
Akkaya (2012)	NI	NI	PN	SOME CONCERNS	UNPREDICTABLE
Forster (1982)	NI	NI	PN	SOME CONCERNS	UNPREDICTABLE
Goodwin (2003)	Y	NI	PN	SOME CONCERNS	UNPREDICTABLE
Hadley (2019)	NI	NI	NI	SOME CONCERNS	UNPREDICTABLE
Kirnap (2005)	NI	PN	PN	SOME CONCERNS	UNPREDICTABLE
Kelln (2009)	PY	PN	PN	LOW RISK	NA
Moffet (1994)	NI	NI	NI	SOME CONCERNS	UNPREDICTABLE
Vervest (1990)	NI	NI	NI	SOME CONCERNS	UNPREDICTABLE

© 2021 Nutarelli S et al. *JAMA Network Open.*

eTable 3. GRADE evidence profile

Certainty assessment							№ of patients		Effect		Certainty	Importance
№ of studies	Study design	Risk of bias	Inconsistency	Indirectness	Imprecision	Other considerations	[standard rehabilitation]	[home-based program]	Relative (95% CI)	Absolute (95% CI)		
**Lysholm score SHORT-TERM (follow up: mean 1-3 months; assessed with: PROs; Scale from: 0 to 100)**												
5	randomised trials	serious [a]	not serious	not serious	serious [b]	publication bias strongly suspected [c]	121	112	-	MD **8.64 SD lower** (15.14 lower to 2.13 lower)	⊕◯◯◯ VERY LOW	CRITICAL
**Lysholm score MID-TERM (follow up: mean 6 months; assessed with: PROs; Scale from: 0 to 100)**												
2	randomised trials	serious [a]	not serious	not serious	serious [b]	publication bias strongly suspected [c]	62	66	-	MD **4.78 SD lower** (9.98 lower to 0.42 higher)	⊕◯◯◯ VERY LOW	CRITICAL
**IKDC score SHORT-TERM (follow up: mean 4-6 weeks; assessed with: PROs; Scale from: 0 to 100)**												
2	randomised trials	serious [a]	not serious [d]	not serious [e]	serious [b]	publication bias strongly suspected [c]	62	66	-	MD **6.73 SD lower** (38.15 lower to 24.69 higher)	⊕◯◯◯ VERY LOW	IMPORTANT
**Knee Flexion SHORT-TERM (follow up: mean 4-6 weeks; assessed with: Goniometer)**												
4	randomised trials	serious [a]	serious [f]	not serious	serious [b]	publication bias strongly suspected [c]	110	92	-	MD **7.40 SD lower** 15.12 lower to 0.32 higher)	⊕◯◯◯ VERY LOW	IMPORTANT
**Knee Extension SHORT-TERM (follow up: mean 4-6 weeks; assessed with: Goniometer)**												
2	randomised trials	serious [a]	not serious	not serious	serious [b]	publication bias strongly suspected [c]	46	30	-	MD **0.55 SD higher** (0.07 lower to 1.18 higher)	⊕◯◯◯ VERY LOW	IMPORTANT
**Single Hop test SHORT-TERM (follow up: mean 4-7 weeks; assessed with: Tape measure)**												
2	randomised trials	not serious	not serious	not serious	serious [b]	publication bias strongly suspected [c]	54	50	-	MD **13.88 SD lower** (30.23 lower to 2.47 higher)	⊕⊕◯◯ LOW	IMPORTANT
**Vertical Hop test SHORT-TERM (follow up: mean 4-7 weeks; assessed with: Tape measure)**												
2	randomised trials	not serious	not serious	not serious	serious [b]	publication bias strongly suspected [c]	54	50	-	MD **3.25 SD lower** (6.20 lower to 0.29 lower)	⊕⊕◯◯ LOW	IMPORTANT

CI: Confidence interval; MD: Mean Difference; Explanations: a. A proportion of information is from studies at high RoB - likely to seriously alter results; b. The combined sample size is insufficient; c. Small studies; d. I^2=60% e. Rated 'NOT SERIOUS' even if in the Kelln et al. (2009) study the intervention group provided treatment wasn't a 'full' standard post-meniscectomy PT program but stationary biking alone (because of the PT supervision); f. I^2=91

© 2021 Nutarelli S et al. *JAMA Network Open.*

eReferences

References of the papers excluded after full text review

1. di Paola J. Disability, impairment, and physical therapy utilization after arthroscopic partial meniscectomy in patients receiving workers' compensation. *Journal of Bone and Joint Surgery - Series A*. 2012;94(6):523-530. doi:10.2106/JBJS.K.00076 (reason for the exclusion: control group with no home exercise program only)
2. Jokl P, Stull PA, Lynch JK, Vaughan V. Independent home versus supervised rehabilitation following arthroscopic knee surgery-A prospective randomized trial. *Arthroscopy: The Journal of Arthroscopic and Related Surgery*. 1989;5(4):298-305. doi:10.1016/0749-8063(89)90145-X (reason for the exclusion: incomplete data, original data no longer available)
3. Koutras G, Pappas E, Terzidis IP. Crossover training effects of three different rehabilitation programs after arthroscopic meniscectomy. *International Journal of Sports Medicine*. 2009;30(2):144-149. doi:10.1055/s-2008-1038843 (reason for the exclusion: non-randomized trial)
4. Birch NC. Anti-inflammatory drug therapy after arthroscopy of the knee. A prospective, randomised, controlled trial of diclofenac or physiotherapy. - PubMed - NCBI. Published 1993. Accessed April 19, 2020. https://www.ncbi.nlm.nih.gov/pubmed/8331125 (reason for the exclusion: no outcome to meta-analyze

© 2021 Nutarelli S et al. *JAMA Network Open.*
